# Interspecific Sample Prioritization Can Improve QTL Detection With Tree-Based Predictive Models

**DOI:** 10.3389/fgene.2021.684882

**Published:** 2021-09-06

**Authors:** Min-Gyoung Shin, Sergey V. Nuzhdin

**Affiliations:** Department of Biological Sciences, University of Southern California, Los Angeles, CA, United States

**Keywords:** interspecific, QTL, random forest, gradient boosting, chickpea, machine learning, sample prioritization

## Abstract

Due to increasing demand for new advanced crops, considerable efforts have been made to explore the improvement of stress and disease resistance cultivar traits through the study of wild crops. When both wild and interspecific hybrid materials are available, a common approach has been to study two types of materials separately and simply compare the quantitative trait locus (QTL) regions. However, combining the two types of materials can potentially create a more efficient method of finding predictive QTLs. In this simulation study, we focused on scenarios involving causal marker expression suppressed by *trans-*regulatory mechanisms, where the otherwise easily lost associated signals benefit the most from combining the two types of data. A probabilistic sampling approach was used to prioritize consistent genotypic phenotypic patterns across both types of data sets. We chose random forest and gradient boosting to apply the prioritization scheme and found that both facilitated the investigation of predictive causal markers in most of the biological scenarios simulated.

## Introduction

In agriculture, one of the most prominent hurdles to overcome has been the development of climate-resilient plants (Muñoz-Amatriaín et al., 2017; Narayana and von Wettberg, 2020; Sokolkova et al., 2020; von Wettberg et al., 2020). The speed and magnitude of worldwide climate change necessitate the accelerated advancement of modern crops (Laderach et al., 2011; Joyce and Rehfeldt, 2013; Chen et al., 2015). To do that, an important step is the investigation of the genetic characteristics of modern crops that have been resistant to improvement, and such an investigation can be accelerated by using advanced technologies. Fortunately, the advent of modern sequencing technology has made it possible to investigate genomes on a finer scale than before (Stich and Melchinger, 2010; Narayana and von Wettberg, 2020). Currently, we can utilize genome-scale sequencing technologies to assemble genomes, locate target genes, and identify genes that are associated with particular traits of interest very efficiently. In agriculture, modern sequence technologies can gain synergetic efficacy when combined with modern breeding systems used to fine-map quantitative trait locus (QTL) regions. For instance, MAGIC and Nested Association Mapping (NAM) are breeding systems that aim to find QTL regions with much finer scale by using multiple parental lines to increase genomic variations (Cavanagh et al., 2008; Kump et al., 2011; Tian et al., 2011; Song et al., 2017; Narayana and von Wettberg, 2020).

In addition to advanced sequencing technologies and breeding systems, interspecific hybrid approaches have been crucial to agricultural advancements (Singh et al., 2013; Alvarez and Guzmán, 2018; von Wettberg et al., 2018; Moenga et al., 2020). Interspecific hybrid is a method involving breeding wild species and cultivar species. Wild species often have genetic information that cultivar species have lost due to the long domestication cycle and genetic bottle neck (von Wettberg et al., 2018; Moenga et al., 2020; Narayana and von Wettberg, 2020). The lost genetic information frequently involves traits like environmental stress resistance and disease resistance (Nelson et al., 2017; Moenga et al., 2020). Using interspecific hybrid, we can investigate wild genetic variations that are associated with important traits and locate causal QTLs by performing association analysis, an application that has been used in staple crop studies. For example, in a 2019 chickpea study, QTLs associated with chickpea germination, flowering duration, and bean characteristics were found using NAM materials developed by crossing cultivar chickpeas and wild chickpea plants collected from Turkey (Warburton et al., 2017; Osorio-Guarín et al., 2019; Shin et al., 2019).

As genetic materials become more advanced, novel methodological approaches are being used in analysis of interspecific hybrid plants. Denser markers allow utilizing single-marker based regression approaches for hybrid plants. Predictive methods such as parametric gBLUP enable marker-assisted selection (MAS) or genomic selection (GS) (Gonzalez-Camacho et al., 2018; von Wettberg and Khoury, 2020; von Wettberg et al., 2020). Moreover, machine learning approaches have been introduced as promising alternatives to parametric predictive approaches when finding QTLs that can be used for future breeding schemes (Chlingaryan et al., 2018; Gonzalez-Camacho et al., 2018; Mittrapiyanuruk and Charoen-Ung, 2018). Not only can machine learning potentially overcome the issues with a relatively small sample size to marker number, it is also useful in capturing the nonlinear form of relationships between marker allelic dosage and phenotype variation (Desta and Ortiz, 2014; Qutrio Baloch et al., 2020). Among various machine learning approaches, random forest and gradient boosting methods are especially effective tree-based methods. These methods build multiple small predictive decision trees to make the final prediction, making them more powerful than prediction methods that use only a single model. Another advantage of these methods is that they can rank markers based on marker contribution to trait prediction (Genuer et al., 2010; Mittrapiyanuruk and Charoen-Ung, 2018; Shah et al., 2019; Shin et al., 2019).

**FIGURE 1:**
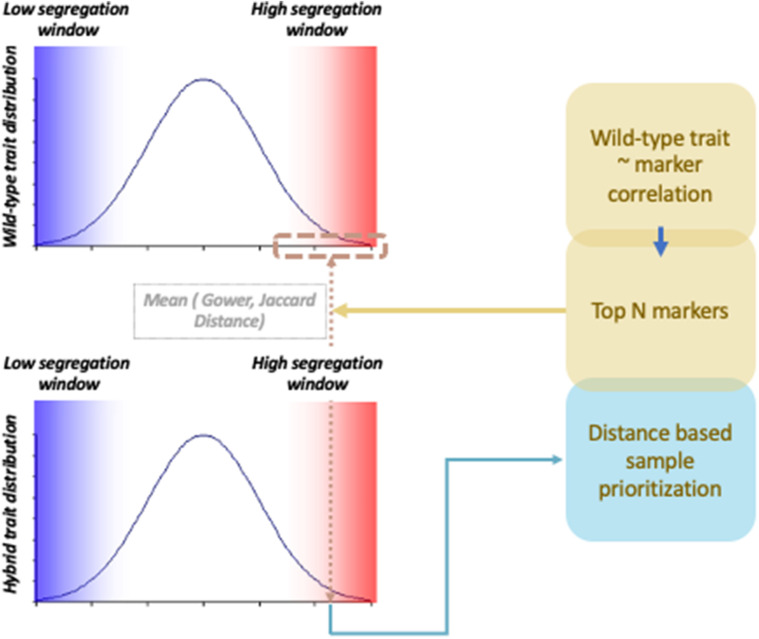
Probabilistic sample prioritization scheme applied to tree-based prediction models.

To compare association signals from wild-type and hybrid materials, scientists analyze two materials independently to investigate shared QTLs. However, by integrating information coming from two different data types, the chance of finding QTLs can be increased. Integration of information can be particularly useful when wild-type genetic regions and cultivar genetic regions have epistatic interactions. *Trans-*regulation of QTLs has been commonly found in interspecific hybrid studies (Heidt et al., 2013; Santos et al., 2015; Gould et al., 2018). For instance, in an *Arabidopsis* study, miR163 was found to be a negative regulator against pathogen/herbivore resistance mechanisms, but to be inactive in *Arabidopsis arenosa* (Ng et al., 2011). By studying the allotetraploid hybrid of the two specifies, the study found that miR163 is repressed by *trans-*regulators. Association signals hidden by *trans-*regulated suppression of phenotype expression can be potentially rescued if we properly prioritize samples with consistent phenotype–genotype correlation across different data types. This can be achieved by investigating samples that share genetic and phenotypic similarity between hybrid and wild-type materials (Figure 1). In this study, hybrid samples were weighted by a distance measure that captures proximity between hybrid samples and wild-type samples using a probabilistic random sampling approach. Two machine learning methods, random forest and gradient boosting, were applied, and their performances were compared with and without the weighting scheme using two different distance measure parameters. The three types of genetic data used were wild-type chickpea, Bari hybrid chickpea, and Egil hybrid chickpea, and phenotypes were simulated based on hybrid materials. The results show that weighted gradient boosting models performed better than unweighted gradient boosting models on all data sets and that weighted random forest performed better than unweighted random forest models in Bari chickpea in large-effect-size scenarios with specific parameter settings. The results suggest that combining information from hybrid and wild-type materials generally performs better in detecting *trans-*downregulated signals in hybrid materials than investigating hybrid material alone.

## Results

### Bari Causal Marker Prediction Efficacy in Random Forest Models

**FIGURE 2:**
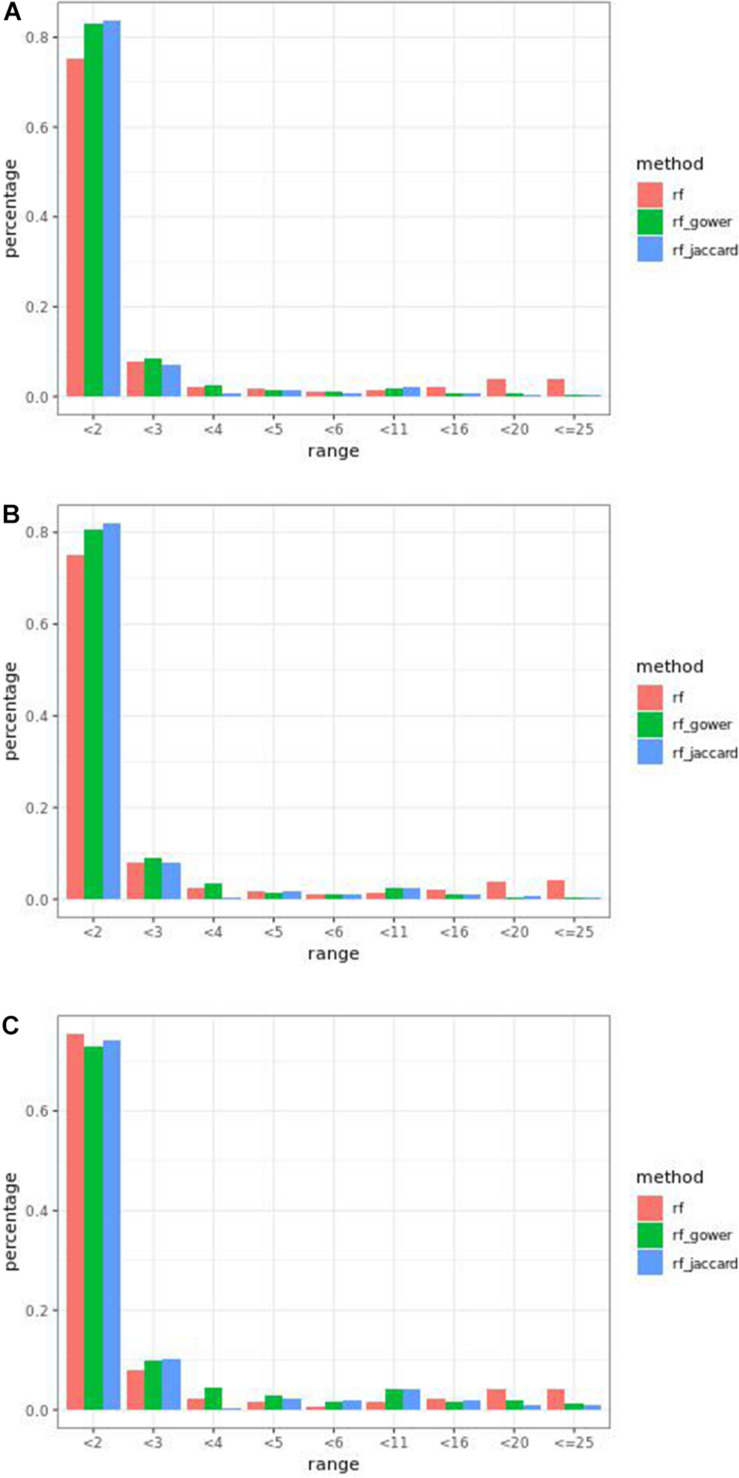
Bari small effect size scenario causal marker detection rates from random forest models, across different rank intervals. The hybrid trait segregation threshold was 5% in panel **(A)**, 10% in panel **(B)**, and 25% in panel **(C).**

Overall, weighted random forest models performed better than unweighted random forest models in finding causal markers (Figure 2A). The only exception was when effect sizes were small and hybrid trait segregation threshold was high. When the threshold was 25%, unweighted prediction models detected causal markers in the first rank bin at a rate of 75%, while weighted prediction models detected causal markers in the same bin at a rate of 73–74% (Figure 2C). The overall mean percentage difference between weighted prediction models and unweighted prediction models in the “rank < 2” interval was 5%.

**FIGURE 3:**
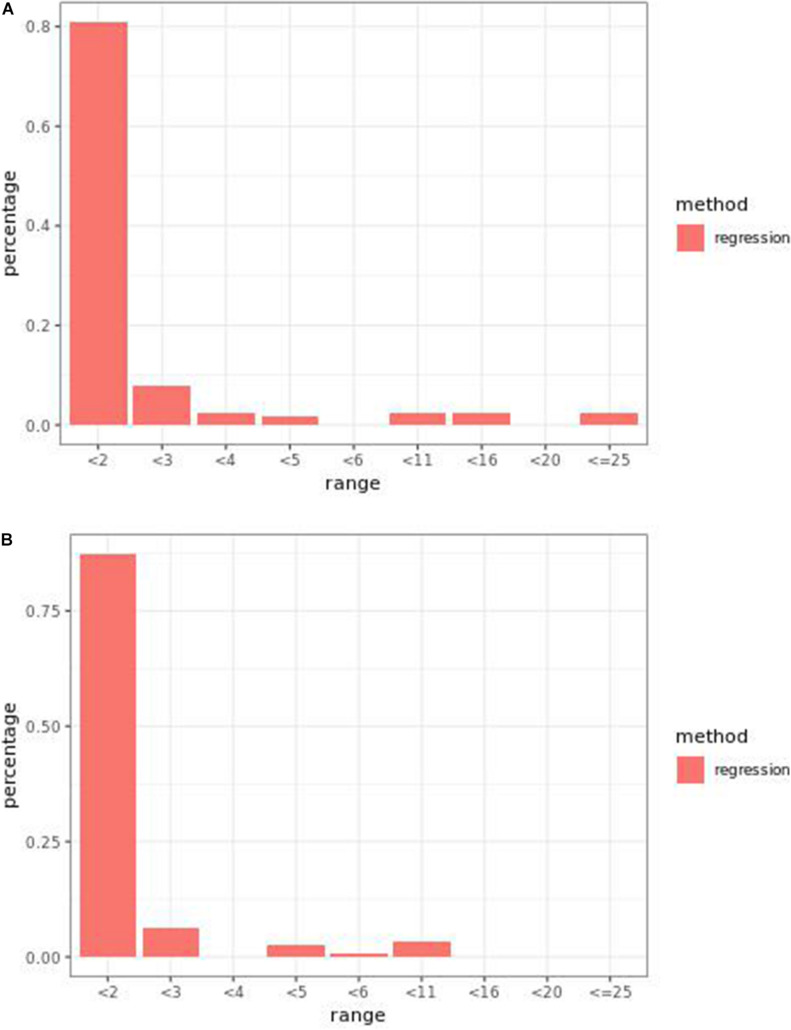
Bari **(A)** small-effect-size scenario and **(B)** large-effect-size scenario causal marker detection rates across different rank intervals in regression models.

Regression models ranked causal markers as the top markers at 81 and 87%, in low-effect-size and large-effect-size scenarios, respectively (Figures 3A,B). When effect sizes were small and hybrid trait segregation thresholds were 5 and 10%, weighted prediction models performed the same as or better than regression models in the first marker rank bin, with detection rates ranging from 81 to 84% (Figures 2A,B). In high-effect-size scenarios, weighted prediction models always performed better than regression models by ranking causal markers in the first marker bin at a minimum rate of 89%.

**FIGURE 4:**
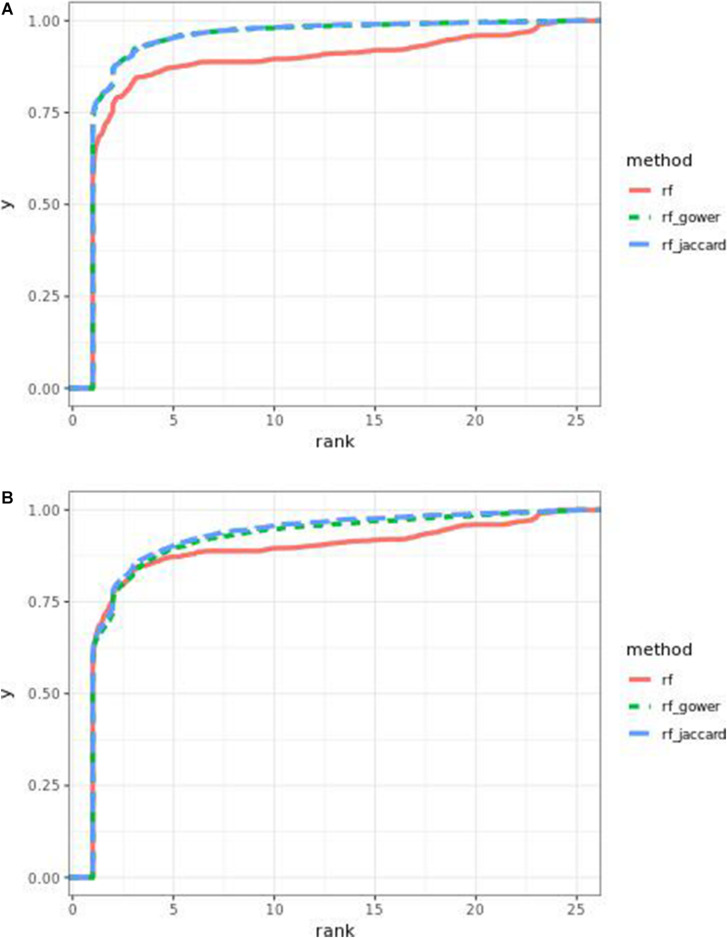
Cumulative Bari small-effect-size scenario causal marker detection rates from random forest models. The hybrid trait segregation threshold was 5% in panel **(A)** and 25% in panel **(B)**.

Performance of weighted prediction models was sensitive to the hybrid trait segregation threshold in low-effect-size scenarios (Figure 4). In the accumulative distribution of causal marker ranks at the hybrid trait segregation threshold of 5%, the 80% quantile coincided with marker rank 1.5, the 90% quantile coincided with the marker rank from 2.7 to 2.8, and the corresponding marker rank range was from 2 to 2.5 at the 80% quantile and from 3 to 5.3 at the 90% quantile at other thresholds.

The overall performance of Jaccard distance-based weighted models and Gower distance-based weighted models was similar in different effect size scenarios and parameter settings. The largest performance difference was 2% at the wild-type trait segregation threshold of 20% in low-effect-size scenarios.

The percentage range of causal markers ranked in “rank < 2” ranged between 73 and 84% in low-effect-size scenarios and 86 to 93% in high-effect-size scenarios. The best performance of prediction models in the first rank bin was reached in high-effect-size scenarios when the hybrid trait segregation threshold was 5%.

### Bari Causal Marker Prediction Efficacy in Gradient Boosting Models

Weighted gradient boosting models consistently performed better than unweighted gradient boosting models. In the “rank < 2” interval, the overall average percentage difference between weighted prediction models and unweighted prediction models was 11%, which is 6% higher compared to random forest results.

**FIGURE 5:**
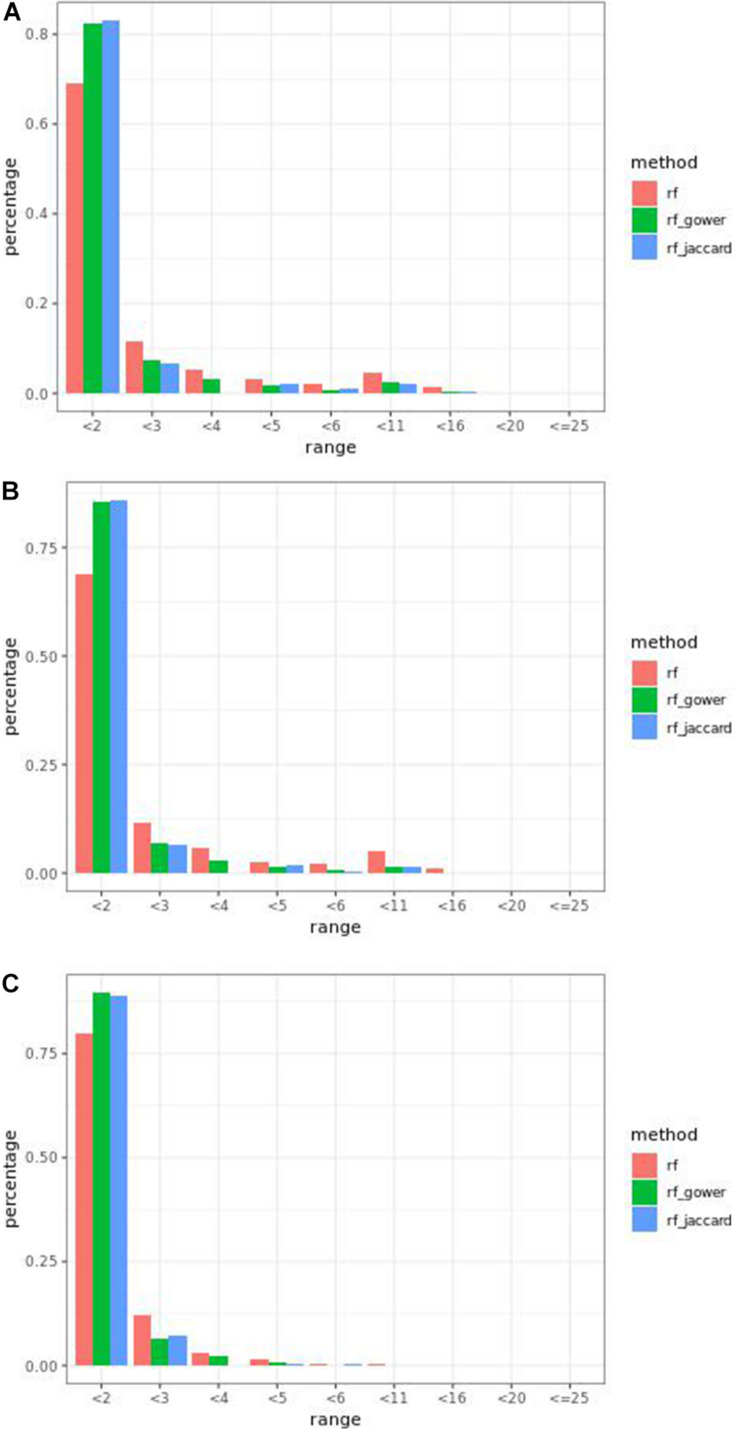
Bari causal marker detection rates across different rank intervals in gradient boosting models. **(A)** The top marker cutoff of 5 in the small-effect-size scenario, **(B)** the hybrid trait segregation threshold of 5% in the small-effect-size scenario, and **(C)** the hybrid trait segregation threshold of 5% in the large-effect-size scenario.

Overall, at least one of the weighted models performed the same as or better than regression models in the first rank bin with few exceptions. In small-effect-size scenarios, weighted models performed better than regression models only when the top marker cutoff was 5 (Figure 5A), while in the same effect size scenarios, weighted models performed the same as or better than regression models when the hybrid trait segregation threshold was less than 20% (Figure 5B). The percentage range of the causal markers ranked as “rank < 2” ranged from 67 to 86% in small-effect-size scenarios and 79 to 89% in large-effect-size scenarios.

**FIGURE 6:**
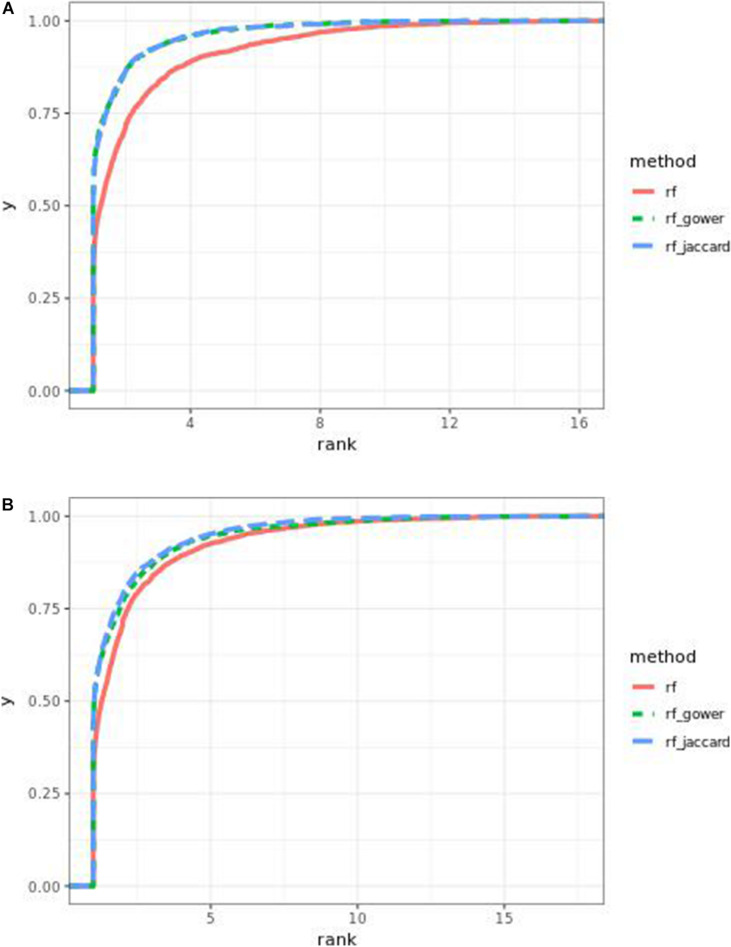
Cumulative Bari small-effect-size scenario causal marker detection rates in gradient boosting models. The hybrid trait segregation threshold was 5% in panel **(A)** and 25% in panel **(B)**.

As observed in random forest results, the weighted model performance was sensitive to the hybrid trait segregation threshold when effect sizes were small (Figure 6). In the accumulative distribution of causal marker ranks, the 80% quantile coincided with the marker rank from 1.6 to 1.7 and the 90% quantile coincided with the marker rank from 2.3 to 2.8 at a threshold below 15%. The corresponding marker rank range was from 1.8 to 2.2 at the 80% quantile and from 3 to 3.5 at the 90% quantile at a higher threshold.

The best performance of prediction models in “rank < 2” was 90%, in large-effect-size scenarios. In addition, at three-parameter settings in large-effect-size scenarios, the performance reached 89%. The best performance was found at a hybrid trait segregation threshold of 5% in large-effect-size scenarios (Figure 5C). Performance of 89% was achieved at a hybrid trait segregation threshold of 10%, at a top marker threshold of 5, and at a wild-type trait segregation threshold of 20%.

### Egil Causal Marker Prediction Efficacy in Random Forest Models

**FIGURE 7:**
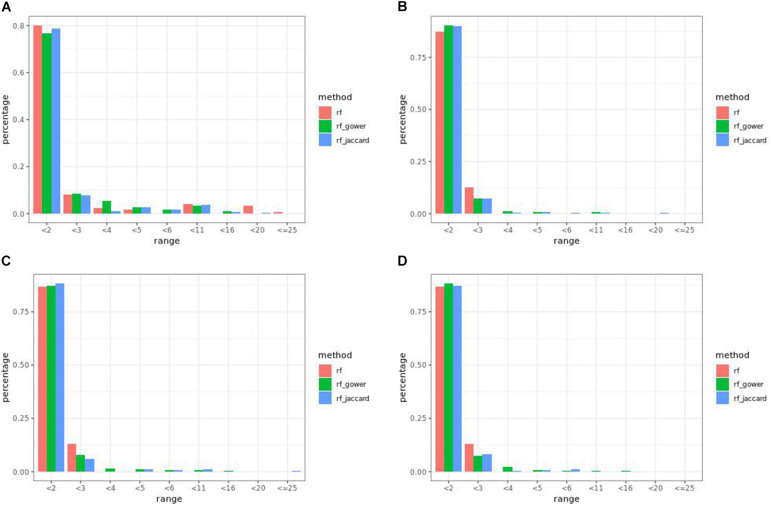
Egil causal marker detection rates across different rank intervals in random forest models. **(A)** Small-effect-size scenarios at the hybrid trait segregation threshold of 5%. **(B)** Large-effect-size scenarios at the hybrid trait segregation threshold of 5%. **(C)** Large-effect-size scenarios at the wild-type trait segregation threshold of 5%. **(D)** Large-effect-size scenarios at the top marker cutoff of 15.

**FIGURE 8:**
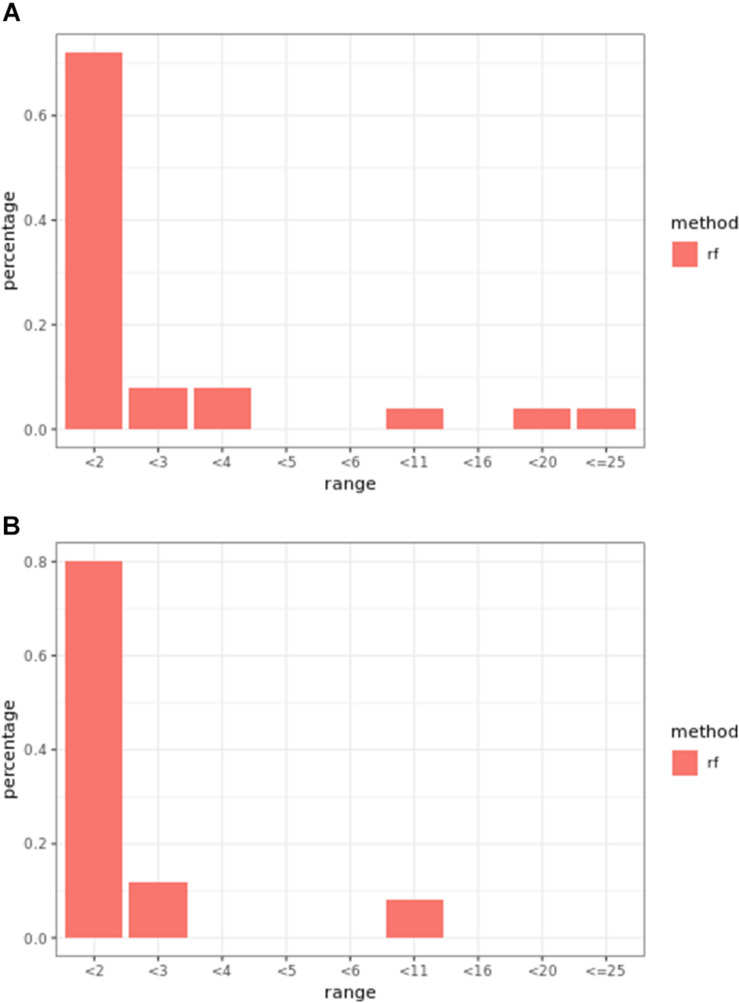
Egil **(A)** small-effect-size scenario and **(B)** large-effect-size scenario causal marker detection rates across different rank intervals in regression models.

In many parameter settings, unweighted random forest models performed better than weighted random forest models in finding causal markers (Figure 7A). Cases in which weighted random forest models performed better than unweighted random forest models in the “rank < 2” interval were found in large-effect-size scenarios. In particular, weighted models outperformed unweighted models in all five types of hybrid trait segregation thresholds (Figure 8B) and at wild-type trait segregation thresholds less than 20% (Figure 7C). Weighted models performed better than unweighted models at all different top marker cutoffs except for the cutoff of 10 in large-effect-size scenarios (Figure 7D). The average difference between weighted model and unweighted model performances was 4%.

At least one of the prediction models ranked causal markers as the top marker with a higher percentage than regression models in all effect size scenarios and parameter settings (Figures 7, 8). The percentage of causal markers ranked in the first rank bin by regression models was 72% in low-effect-size scenarios and 80% in high-effect-size scenarios, while the percentage range of causal markers ranked as top markers by prediction models ranged from 66 to 80% in low-effect-size scenarios and 87 to 91% in high-effect-size scenarios.

**FIGURE 9:**
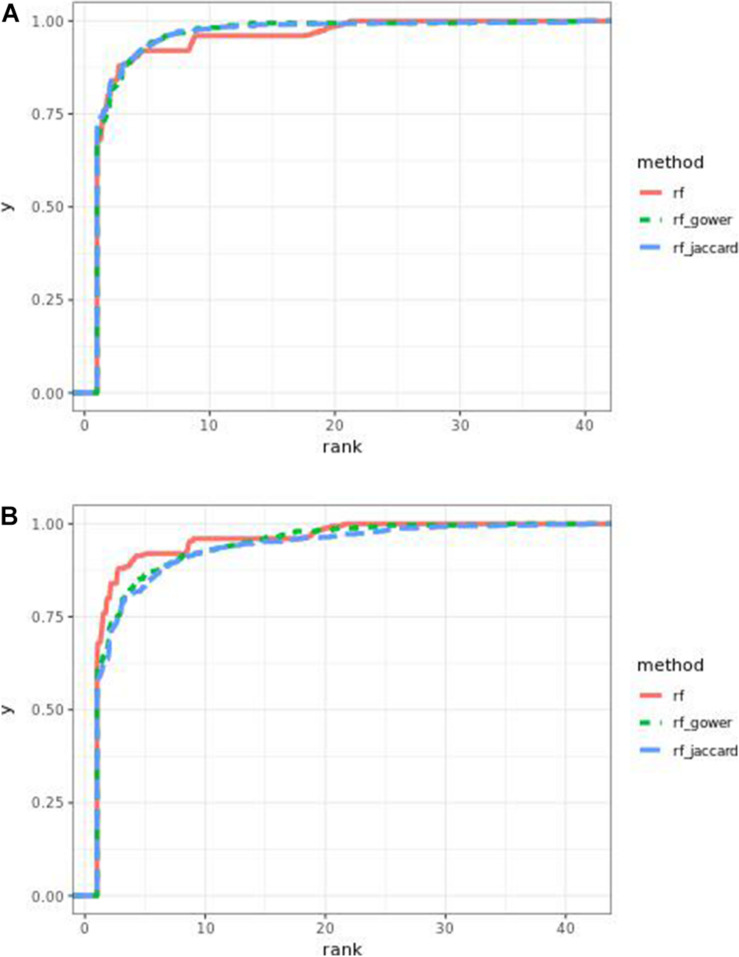
Cumulative Egil small-effect-size scenario causal marker detection rates in random forest models. The hybrid trait segregation threshold was 5% in panel **(A)** and 25% in panel **(B)**.

The performance of weighted prediction models was sensitive to the hybrid trait segregation threshold in low-effect-size scenarios (Figure 9). Weighted prediction models performed best at a threshold of 5% based on causal marker rank quantiles. In the accumulative distribution of causal marker ranks, the 80% quantile coincided with the marker rank 2, and the 90% quantile coincided with the marker rank from 3.9 to 4 at the threshold of 5%, while the corresponding marker rank range was from 3 to 3.4 for the 80% quantile and from 5 to 9.2 for the 90% quantile in other thresholds.

The best performance in “rank < 2” was achieved by Gower distance-based weighted models at a hybrid trait segregation threshold level of 10% in high-effect-size scenarios. The second-best performance in the same rank bin was from the same type of model at a hybrid trait segregation threshold level of 5% in high-effect-size scenarios. In general, Jaccard distance-based weighted prediction models performed similarly to Gower distance-based weighted prediction models.

### Egil Causal Marker Prediction Efficacy in Gradient

**FIGURE 10:**
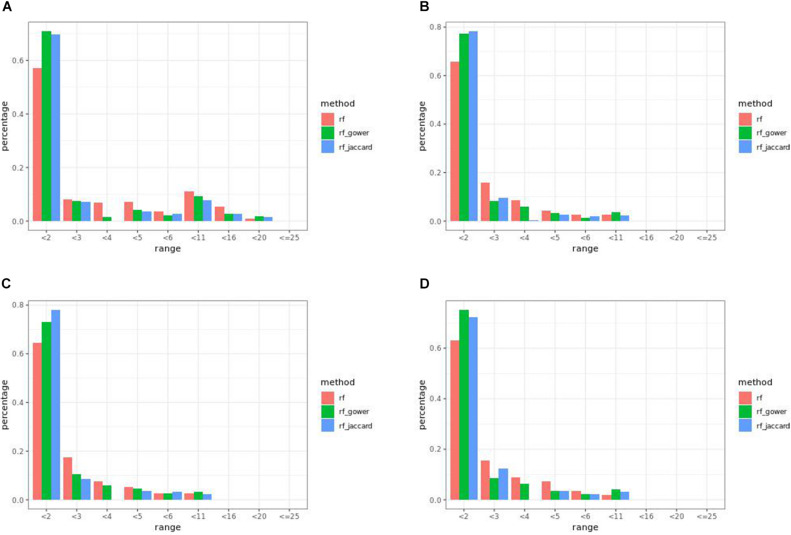
Egil causal marker detection rates across different rank intervals in gradient boosting models. **(A)** Small-effect-size scenarios at the hybrid trait segregation threshold of 5%. **(B)** Large-effect-size scenarios at the hybrid trait segregation threshold of 5%. **(C)** Large-effect-size scenarios at the top marker cutoff of 20 and **(D)** cutoff of 5.

Weighted gradient boosting models consistently performed better than unweighted gradient boosting models (Figure 10). The overall average percentage difference between weighted prediction models and unweighted prediction models in the “rank < 2” interval was 8%, while the difference was 4% in random forest results.

Regression models always performed better than prediction models (Figures 7, 10). The percentage range of causal markers ranked as top markers ranged from 56 to 71% for low-effect-size scenarios and 63 to 78% for high-effect-size scenarios.

**FIGURE 11:**
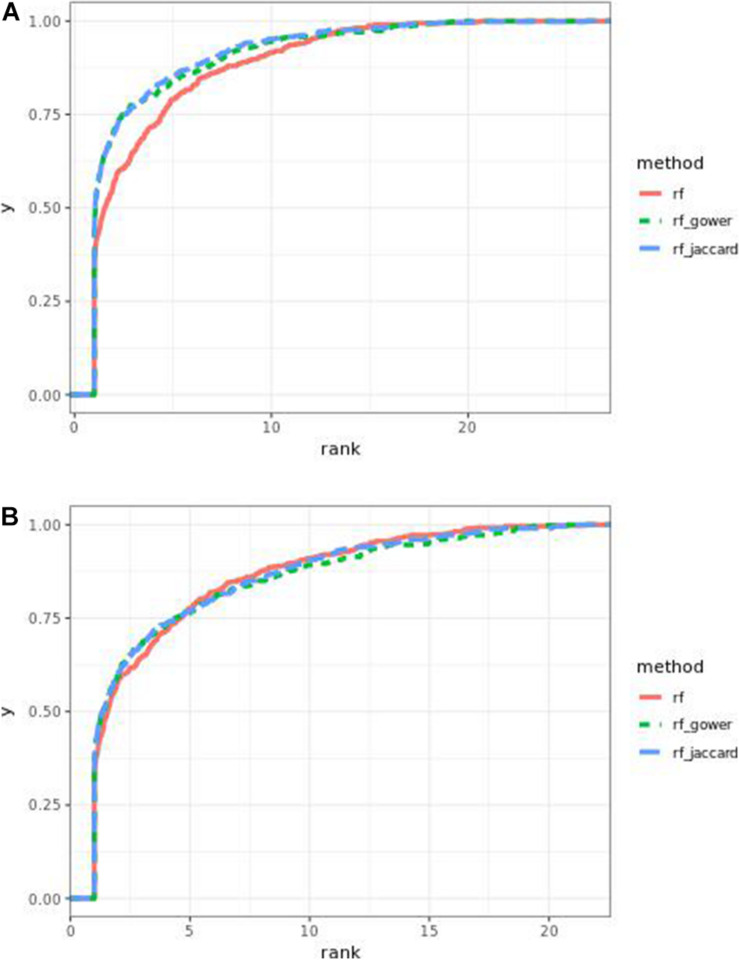
Cumulative Egil small-effect-size scenario causal marker detection rates in gradient boosting models. The hybrid trait segregation threshold was 5% in panel **(A)** and 25% in panel **(B)**.

The performance of weighted prediction models was sensitive to the hybrid trait segregation threshold in low-effect-size scenarios as observed in Bari and Egil random forest results (Figure 11). In the accumulative distribution of causal marker ranks at the hybrid trait segregation threshold of 5%, the 80% quantile coincided with the marker rank from 3.6 to 4, and the 90% quantile coincided with the marker rank from 7 to 7.5, while the corresponding marker rank range was from 4.6 to 6 at the 80% quantile and from 8.7 to 11 at 90% quantile in the other thresholds.

The overall performance of Jaccard distance-based weighted models and Gower distance-based weighted models was similar in most of effect size scenarios and parameter settings, and in some cases, the difference between the two approaches was more than 2%. For instance, in large-effect-size scenarios with a top marker cutoff of 20, Jaccard distance-based models performed 5% better than Gower distance-based models in the first rank bin (Figure 10C). On the other hand, in large-effect-size scenarios with a top marker cutoff of 5, Gower distance-based models performed 3% better than Jaccard distance-based models in the same rank bin (Figure 10D).

The highest percentage of causal markers ranked as “rank < 2” was 78%, which was when effect sizes were large and the hybrid trait segregation threshold was 5% and when the top marker cutoff was 20.

## Discussion

Random forest and gradient boosting methods are widely applied powerful machine learning methods (Lubke et al., 2013; Mittrapiyanuruk and Charoen-Ung, 2018; Shin et al., 2019). Although both approaches adapt a tree-based model scheme, their internal logic is different, and the investigation of their performance difference according to different types of data sets can provide valuable information. This study used two different data sets, Bari chickpea and Egil chickpea data, and compared the performance of two machine learning approaches. Additionally, a weighting scheme that prioritizes hybrid samples that share similarity with wild-type samples in terms of genotypic and phenotypic patterns was tested. The biological context of the simulation was confined to a scenario where causal marker effect is suppressed by epistatic interaction in hybrid plants since such a lost signal can take advantage of signals from wild-type materials. This study suggests performance improvement of random forest and gradient boosting methods in identification of causal loci from interspecific hybrid data.

In both datasets, weighted gradient boosting models generally performed better than unweighted gradient boosting models. In the “rank < 2” interval, the performance of weighted gradient boosting models was 11% and 8% better than unweighted gradient boosting models in Bari and Egil data, respectively. For random forest, weighted models performed better in majority parameter settings only with the Bari data. In Bari, for small-effect-size scenarios, the performance of weighted models was better than unweighted models only when the hybrid trait segregation threshold was less than 20%. In Egil, large effect sizes and lower trait segregation thresholds contributed to the improved performance of weighted random forest models. In summary, gradient boosting is more likely to provide consistent results across different data sets and parameters.

In the 80 and 90% quantiles of causal marker ranks, the model performance was sensitive to the hybrid trait segregation threshold. In all cases, the smallest threshold, 5%, showed the smallest marker ranking, indicating that a small hybrid trait segregation window tends to rank causal markers closer to the top markers. Not surprisingly, in the first rank bin, the best performance was found at hybrid trait segregation thresholds of 5 or 10%. However, the parameters wild-type trait segregation threshold and top marker cutoff were not factors that altered the efficacy of the predictive model considerably.

Although the Gower distance and Jaccard distance measurements resulted in different rates of causal marker detection efficacy in a few cases, there was no dramatic difference in the performance of the two measurements. Therefore, it is not expected that the choice of distance metric would be the key factor in changing the performance of the predictive model.

In this study, random forest models achieved the highest percentage of causal marker detection as top markers regardless of whether the samples were weighted or not. However, because random forest models are more sensitive to the data set and effect size, it can be difficult to decide whether to use a weighted or unweighted model based on the characteristics of the data set of interest. In conclusion, a weighted gradient boosting model can be proposed as a method that provides robust causal marker ranking efficiency across different data and parameter settings.

## Materials and Methods

### Random Forest

Random forest is an ensemble method composed of multiple decision trees. It takes subsets of samples, which is called bagging, to build each decision tree and uses aggregate predictions from multiple decision trees to make a final prediction. This subsampling strategy is known to produce reliable results, balancing variance and bias. In this study, 1,000 decision trees were used, and RMSE was chosen as the loss function. To estimate variable importance, a permutation-based method was applied. Permutation-based variable importance reflects the change of prediction accuracy measured from out-of-bag samples, which are the samples that were not included in decision tree training. The change of prediction is the difference of prediction error from permuted data where a target variable was permuted and prediction error from non-permuted data. In this study, variable importance was measured from 100 independent iterations, and the average rank was reported.

### Gradient Boosting

Another type of ensemble learner, gradient boosting, uses multiple weak learners which contribute to the final prediction. The difference between gradient boosting and random forest is that the former focuses on residual values of prediction performed on the previous step. This concept can be formulized as follows:

$${\hat{y}}_{t}={\hat{y}}_{t-1}+\eta h\left(y,{\hat{y}}_{t-1}\right)$$

where ${\hat{y}}_{t}$ is a predicted value at step *t*, η is a learning rate, and *h* is the negative gradient of the loss from the given loss function, which is the squared error in this study. Each step aims to reduce the loss by taking into account the gradient of the previous loss function. In this study, 0.3 was used as an η value. To estimate variable importance from gradient boosting, gain was measured. Gain is a measurement that represents the relative contribution of each marker to the model, and it is calculated using increased score as a result of a new branch. In this study, variables were ranked based on gain in 100 independent iterations, and the average rank was reported.

### Independent Marker Filtering

Tree-based methods provide variable importance measures which can be used to prioritize markers. However, when there is a correlation between markers, the returned results can be less accurate because variance importance can be unevenly assigned to one of the correlated markers. To alleviate this issue, markers were filtered based on pairwise correlation coefficients before performing prediction analysis. To select markers with low pairwise correlation coefficients, correlation between all pairwise markers was calculated first. Based on calculated correlation coefficients, adjacency networks were constructed. To do this, each marker was assigned to each node in a network, and a pairwise relationship with a correlation coefficient larger than 0.7 was used to connect a pair of nodes that correspond to the pair of markers. Assignment of networks was carried out using the R package igraph (Csardi and Nepusz, 2005); then one representative marker from each connected component was selected. To assist causal marker rank assessment, if a causal marker was a member of a connected component, the causal marker was selected as a representative marker, and otherwise, a random marker was selected. In total, 125 markers were selected from Bari data, and 25 markers were selected from Egil data.

### Phenotype Simulation

To simulate phenotype, causal markers were chosen as the intersect of hybrid markers and wild-type markers. We emphasize that our analysis does not and cannot establish a causality; rather, it relies on prior knowledge of causal effects and observes how frequently they are in fact detected. To select causal markers with higher heterozygosity in wild-type materials, a minor allele frequency ratio threshold 1.5 was used. After applying the filtering and selecting independent markers according to the procedure described in section “Independent marker filtering,” a total of 125 markers were selected from 2,400 Bari markers, and 25 markers were selected from 2,132 Egil markers. To simulate phenotypes with different effect sizes, variance explained values of 0.4 and 0.8 were chosen. Phenotypes were simulated based on allelic dosage of a causal marker, in an additive manner, and random noise was introduced to adjust effect sizes. Random noise was simulated by using the R package rmvnorm (Genz et al., 2009) with an n × n identity matrix, where n is the number of samples, and random variance was sampled. To simulate the effect of epistatic *trans-*downregulation, one random marker was selected, and the phenotype value was suppressed when the random marker had an allele dosage bigger than zero.

### Weighted Models

In addition to standard random forest and gradient boosting models, weighted models are applied to prioritize samples with consistent genetic and phenotypic patterns across wild-type materials and hybrid materials. Prioritization was achieved by applying a probabilistic sampling scheme where probability reflects the importance of each sample. First, markers to focus were selected from wild-type materials. The correlation of wild-type phenotype and allelic dosage of each marker was calculated, and the top correlated markers were selected. To verify the impact of the number of selected markers, different numbers of top markers were applied, and the corresponding performance of prediction was tested. The varied numbers of markers were 5, 10, 15, and 20. Next, wild-type samples were filtered based on trait segregation level. Samples with segregating traits were chosen, applying different quantiles to assess the impact of the number of chosen wild-type samples. The choices of quantiles were 5, 10, 15, 20, and 25%. Then, hybrid samples were filtered using the same scheme using five types of quantiles. Filtered hybrid samples were prioritized based on average similarity with filtered wild-type samples. Similarity between each hybrid sample and filtered wild-type samples was measured using two distance metrics, Gower distance and Jaccard distance, which can be applied to measure the distance between variables with discrete features. The Gower distance is defined as follows (Gower, 1971):

$$S_{ij}=\frac{\sum_{k=i}^{n}s_{ijk}\delta_{ijk}}{\sum_{k=i}^{n}\delta_{ijk}}$$

where *i* and *j* are samples, *k* is SNP, and *s* is the contribution score:

$$s_{ijk}=\frac{\left|x_{ik}-x_{jk}\right|}{R_{k}}$$

where *x*_*ik*_ is the dosage of SNP *k* in sample *i* and *R_k_* is the dosage range of SNP *k*.

δ_*ijk*_ is a weight function that is zero when SNP *k* is invalid for one or more samples. Jaccard distance is defined as follows:

$$J_{ij}=1-\frac{\sum_{k=i}^{n}I_{ijk}}{\sum_{k=i}^{n}I_{k}}$$

where *I*_*ijk*_ is 1 only when SNP *k* has the same dosage in sample *i* and *j* and *I_k_* is 1 when at least one of the samples has non-zero dosage in SNP *k*.

Averaged distance was used to assign random sampling probability to each hybrid sample. In other words, filtered hybrid samples that share a high similarity with filtered wild-type samples were designed to be sampled with higher probability. Probabilistically, random samples were plugged into random forest and gradient boosting models.

### Regression

Regression analysis was performed to assess the rank of causal markers using linear regression models. The analysis was performed using the genome-wide association analysis tool PLINK.

### Genetic Materials

#### Chickpea Materials

The 143 wild chickpea samples used in this study were a subset of chickpea samples collected in Turkey, which is known as the origin of chickpea (von Wettberg et al., 2018). The Bari and Egil data used in this study are subsets of the 2,521 F2 hybrid chickpea materials crossed between 20 wild-type parent lines and the early flowering parent ICCV96029. After further filtering based on FT locus to prevent the confounding effect of segregating phenology linked to that locus, 284 F2 lines were selected (Shin et al., 2019). To perform GBS sequencing, restriction enzymes *Hin*dIII and *Nla*III were used, and Illumina HiSeq 4000 was used to generate sequence data. Hybrid genotype data are available online at the National Center for Biotechnology Information under the BioProject umbrella PRJNA353637. Illumina reads were mapped to the *Cicer arietinum* CDC Frontier reference genome using BWA MEM, and variants were called using the GATK pipeline and were filtered using hard filtering parameters: MQ > 37, QD > 24, MQRankSum < 2. The numbers of samples were 143, 140, and 124, in wild-type chickpea, Bari chickpea, and Egil chickpea, respectively, and the corresponding numbers of markers were 1,946, 2,400, and 2,132, respectively.

## Data Availability Statement

The datasets presented in this study can be found in online repositories. The names of the repository/repositories and accession number(s) can be found below: https://www.ncbi.nlm.nih.gov/, PRJNA353637.

## Author Contributions

M-GS contributed to the conception and design of the study, performed the analysis, and wrote the manuscript. SN contributed to the conception and manuscript revision. All authors contributed to the manuscript revision, read, and approved the submitted version.

## Conflict of Interest

The authors declare that the research was conducted in the absence of any commercial or financial relationships that could be construed as a potential conflict of interest.

## Publisher’s Note

All claims expressed in this article are solely those of the authors and do not necessarily represent those of their affiliated organizations, or those of the publisher, the editors and the reviewers. Any product that may be evaluated in this article, or claim that may be made by its manufacturer, is not guaranteed or endorsed by the publisher.
